# Clinical Outcome Prediction in Aneurysmal Subarachnoid Hemorrhage Using Bayesian Neural Networks with Fuzzy Logic Inferences

**DOI:** 10.1155/2013/904860

**Published:** 2013-04-10

**Authors:** Benjamin W. Y. Lo, R. Loch Macdonald, Andrew Baker, Mitchell A. H. Levine

**Affiliations:** ^1^Divisions of Neurosurgery & Critical Care Medicine, St. Michael's Hospital, University of Toronto, 30 Bond Street, 3 Bond Wing, Toronto, ON, Canada M5B 1W8; ^2^Keenan Research Centre of the Li Ka Shing Knowledge Institute of St. Michael's Hospital, University of Toronto, 30 Bond Street, 3 Bond Wing, Toronto, ON, Canada M5B 1W8; ^3^Department of Critical Care, Trauma and Neurosurgery Program, Keenan Research Centre, Li Ka Shing Knowledge Institute, St. Michael's Hospital, Toronto, ON, Canada; ^4^Departments of Anesthesia and Surgery, University of Toronto, 30 Bond Street, 3 Bond Wing, Toronto, ON, Canada M5B 1W8; ^5^Department of Clinical Epidemiology & Biostatistics, and Department of Medicine, Centre for Evaluation of Medicines, St. Joseph's Hospital, McMaster University Toronto, ON, Canada

## Abstract

*Objective*. The novel clinical prediction approach of Bayesian neural networks with fuzzy logic inferences is created and applied to derive prognostic decision rules in cerebral aneurysmal subarachnoid hemorrhage (aSAH). *Methods*. The approach of Bayesian neural networks with fuzzy logic inferences was applied to data from five trials of Tirilazad for aneurysmal subarachnoid hemorrhage (3551 patients). *Results*. Bayesian meta-analyses of observational studies on aSAH prognostic factors gave generalizable posterior distributions of population mean log odd ratios (ORs). Similar trends were noted in Bayesian and linear regression ORs. Significant outcome predictors include normal motor response, cerebral infarction, history of myocardial infarction, cerebral edema, history of diabetes mellitus, fever on day 8, prior subarachnoid hemorrhage, admission angiographic vasospasm, neurological grade, intraventricular hemorrhage, ruptured aneurysm size, history of hypertension, vasospasm day, age and mean arterial pressure. Heteroscedasticity was present in the nontransformed dataset. Artificial neural networks found nonlinear relationships with 11 hidden variables in 1 layer, using the multilayer perceptron model. Fuzzy logic decision rules (centroid defuzzification technique) denoted cut-off points for poor prognosis at greater than 2.5 clusters. *Discussion*. This aSAH prognostic system makes use of existing knowledge, recognizes unknown areas, incorporates one's clinical reasoning, and compensates for uncertainty in prognostication.

## 1. Introduction

Advances in biostatistics and computing in the past several decades have led to creation of different types of clinical outcome prediction models. Three of these include artificial neural networks, fuzzy logic and bayesian analysis [1–3]. These techniques complement classical or frequentist approaches, such as regression analysis.

Artificial neural networks mimic biological neural systems. In biological systems, incoming dendrites collect signals which are fed to the neuron. A signal summation is then sent as a spike of electrical current along an axon, with resultant discharge at the synapse, connecting it to other neurons. Examples of biological neural networks include the human brain and the human retina. Analogous to the biological system, artificial neural networks are made up of a group of input variables which converge on a number of nodes. Nodes are grouped in layers, with interconnection links among themselves. Hidden or latent variables can exist in one or two layers. After processing from different activation functions, output signals are then sent onto output nodes in the network. artificial neural networks assume all or none logic, that is, subjects are regarded as having or not having a diagnosis. Nodes in the neural nets are connected with each other via connection links. Each of these links has an associated weight and activation function. Neural networks are intelligent systems that can learn and change behaviour by themselves as they gain experience. In addition, they also take into account unobservable variables that the researcher is not aware of while designing the neural net.

**Figure 1:**
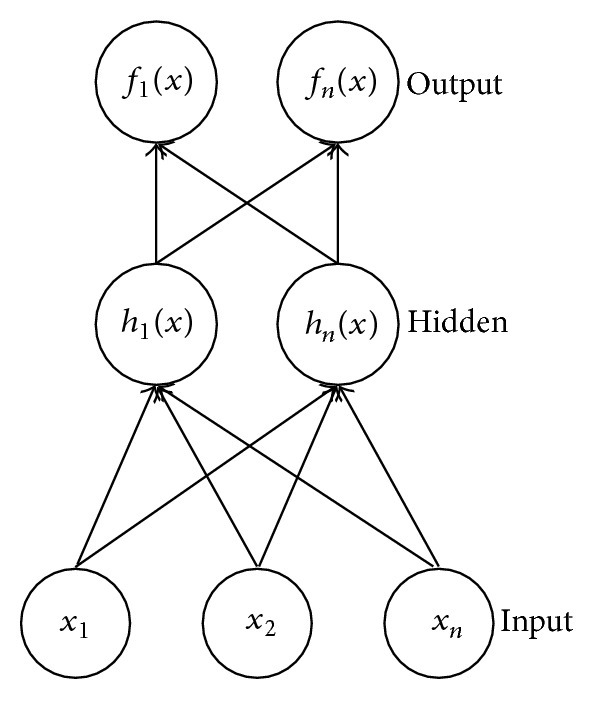
Artificial Neural Network with 3 inputs, 2 hidden nodes and 2 outputs. Figure by B. W. Y. Lo.

Assuming a basic artificial neural network with *x*_*n*_ inputs, *h*_*n*_(*x*) hidden or latent variables (in 1 layer), and *f*_*n*_(*x*) an outputs, using a multilayer perceptron model as illustrated in Figure 1, output is equal to the summation of input to hidden layer, as well as hidden layer to output layer.

If the activation function is the nonlinear hyperbolic tangent function, then,

(1) hj(x)=tanh(aj+∑iuij∗  xi),

where hidden unit *j* = hyperbolic tangent function × (bias term + sum of (weights from input unit *i* to hidden unit *j*  × input unit  *i*)), and,

(2) output=fn(x)=bk+∑jvjkhj(x),

where output = *f*_*n*_(*x*) = bias term + sum of (weights on connection from hidden unit *j* to output unit *k*  ×  hidden unit  *j*).

Bayesian analysis allows the researcher to make use of existing states of knowledge before incorporation of new data. Simply put, it reflects the fact that knowledge is cumulative. Here, existing knowledge is expressed in the form of distributions (such as the normal bell-shaped distribution). The “prior” distribution is then combined with its likelihood of occurrence, forming a posterior probability. The end result (or posterior probability) represents a revised or updated belief after taking new data into account. If there is a lack of existing knowledge on the subject of interest, the researcher can still rely on Bayesian techniques. In this case, vague or uninformed prior probabilities are used.

In the Bayesian approach to artificial neural networks [4], the goal is to find the predictive distribution for target values in the new test case/model, given inputs for that case and inputs/targets in training cases. Here, *p*(*D*) represents the probability of data according to a particular model. It is an integral, representing the summation of all possible parameter values weighted by the strength of belief (as assigned by the researcher) in these parameter values, or

(3) p(D)=∫dθp(D ∣ θ)p(θ).

The probability of a new case/model given existing cases and associated parameters (*θ*, incorporating both weights and biases) is expressed as

(4) p(y3 ∣ x3,(x1,y1),(x2,y2))  =∫dθ  p(y3 ∣ x3,θ)p(θ ∣ (x1,y1),(x2,y2)).

In general, Bayesian neural networks can be expressed as:

(5) p(yn+1 ∣ xn+1,(x1,y1),…,(xn,yn))  =∫dθ  p(yn+1 ∣ xn+1,θ)p(θ ∣ (x1,y1),…,(xn,yn)).

Posterior probability density is proportional to product of prior probability density and its associated likelihood. Likelihood, as explained above, is the product of probabilities of data given parameters (weights and biases) as

(6) L(θ ∣ (x1,y1),…,(xn,yn))=∏i=1np(yi ∣ xi,θ).  

Fuzzy logic is an extension of neural nets, but with the distinct advantage that it can assume functions with any value from 0 to 1, accounting for the entire spectrum of certainty of diagnoses and spectrum of severity of diseases studied. It registers a mild case of a certain disease, as it recognizes grey zones in diagnoses. Another strength of fuzzy logic lies in its explicit knowledge representation; that is, it allows the clinician to explicitly state its inputs, control actions, and outputs. The clinician can also clarify, or defuzzify, the entire process by carrying out crisp control actions, such as adding a cut-off level for prognoses and diagnoses, and trigger thresholds for treatment. By doing so, all actions (verification) in fuzzy logic are accounted for, and optimization is achievable. Thus, fuzzy logic systems are efficient ones. On the other hand, some regard this strength of fuzzy logic as a limitation. Unlike neural nets, where learning is done by the nets themselves as they gain experience from datasets, fuzzy logic systems cannot train themselves. The designer has to derive all action commands (in the form of “if-then” rules) manually, which is labour intensive with large datasets. When the degrees of various problems are combined in an equation, however, the resulting calculation may represent a more accurate prediction of the real data and individual patient.

Bayesian neural networks with fuzzy logic inferences can be represented as follows:

(7) Expected  outcome  of  function(Fuzzy-Bayesian  Neural  Network) =defuzzification  technique  applied  toa  Bayesian  Neural  NetworkE(f)=defuzz[p(yn+1 ∣ xn+1,(x1,y1),…,(xn,yn))]=defuzz[∫dθ  p(yn+1 ∣ xn+1,θ)      ×p(θ ∣ (x1,y1),…,(xn,yn))],

where *defuzz* can be max-min, centroid, left of mean, right of mean, or another defuzzification crisp control action rule.

## 2. Objectives and Relevance

In this paper, we aim to use advanced biostatistical methods to create clinical prognostic decision rules in aneurysmal subarachnoid hemorrhage derived from a large aneurysmal subarachnoid hemorrhage database, which can be tailored to a specific patient population. We explore novel methods that account for existing states of knowledge (Bayesian meta-analysis and regression), complex nonlinear relationships between independent, latent, and dependent variables (artificial neural networks), and grey zones in prognoses (fuzzy Logic decision rules). In fact, the combination of such techniques can represent a novel health research method for clinical outcome prediction applicable to many diseases in medicine.

## 3. Methods

Intracranial aneurysmal subarachnoid hemorrhage affects about 45, 000 individuals in North America annually. Aneurysmal subarachnoid hemorrhage is associated with a mortality rate of at least 45% in the first 30 days following rupture [5, 6]. Apart from the primary neurological injury from the aneurysmal rupture itself, other secondary injury processes can further worsen an individual's neurological condition and eventual clinical outcome. These processes include both neurological processes (such as delayed stroke, rebleeding, brain swelling, vasospasm induced strokes, seizures, and hydrocephalus) and systemic medical complications (such as myocardial infarction, fever, pulmonary edema). and Taken together, these processes can lead to long-term disability. Types of disability include physical, neurocognitive, and psychological impairment [7].

Tirilazad aneurysmal subarachnoid hemorrhage database is used to illustrate prognostic decision principles derived from a combination of techniques from multiple linear regression, artificial neural networks, fuzzy Logic and bayesian analysis. Tirilazad is a 21-aminosteroid compound produced by Pharmacia & Upjohn, Kalamazoo, MI, USA, originally investigated by the University of Virginia Health Sciences Center, as a free radical scavenger for potential treatment of cerebral vasospasm. This medication was investigated in five randomized clinical trials [8–12] involving patients with aneurysmal subarachnoid hemorrhage between 1990 and 1997 in 162 centers from 21 countries across North America, Europe, Australia, New Zealand, and South Africa. Tirilazad was found to have no effect on clinical outcome in patients with aneurysmal subarachnoid hemorrhage. The resultant database from these five studies contains 3550 patients, with its primary outcomes being Glasgow outcome score at 3 months and death from any cause. Glasgow outcome score is a 5-point neurological scale with the following designations: 5: good recovery—normal life activities despite minor deficits, 4: moderate disability—disabled but independent, 3: severe disability—conscious but disabled, 2: persistent vegetative state—unresponsive and speechless, and 1: death [13].

Centers followed strict treatment protocols, and variables had fewer than 5% missing entries. Tirilazad was administered in the intravenous form from day 3 to day 10 after subarachnoid hemorrhage onset. Only one percent of patients were lost to followup.

Patients in each treatment group were managed in a similar manner. Over 85% underwent surgical clipping, with 50% operated within the first 48 hours. Baseline demographics in both treatment and control arms were balanced in terms of gender, age, number of preexisting medical conditions (including hypertension, myocardial infarction, and angina), mean time to treatment, mean admission systolic blood pressure, admission neurological grade, ruptured aneurysm location, and admission amount of blood. These potential confounders were accounted for in statistical analysis. Proportions of patients experiencing vasospasm were similar in different treatment groups, as were the percentages of patients experiencing both neurological and systemic disabilities, including cerebral hemorrhage, cerebral ischemia, second stroke, rebleeding, hydrocephalus, sepsis, pulmonary embolus, brain herniation, pneumonia, and renal insufficiency.

A Cochrane systematic review [14] on the five trials on Tirilazad found no substantial heterogeneity among the trials, and there were no significant differences in adverse events between the treatment group and placebo group. The Tirilazad database represents the largest currently available aneurysmal subarachnoid hemorrhage clinical trial database worldwide.

Derivation of prognostic decision rules comprised of the following investigations: multivariable linear regression (IBM SPSS version 19.0 (Armonk, NY, USA)), artificial neural networks (IBM SPSS), Bayesian regression with uninformed and informed prior likelihoods (WinBUGS 1.4.3), neural networks with Bayesian regularization (MATLAB R2012a (Natick, MA, USA); version R 2.15.2), and creation of fuzzy logic decision rules, with clinical case application.

## 4. Results & Discussions

### 4.1. Multivariable Linear Regression Analysis

Frequentist linear regression was created using IBM SPSS. Included in the analysis were predictor variables from the Tirilazad database, without recoding, renaming, reclassification, or data transformation. Treatment variables were not included in this analysis. Predictor variables included were age, gender, neurological grade, intraventricular hemorrhage, subarachnoid hemorrhage thickness, time to treatment, clinical vasospasm, mean arterial pressure, aneurysm location, prior anticoagulation, eye opening, normal motor response, normal speech, admission angiographic vasospasm, intracerebral hemorrhage, hydrocephalus, prior subarachnoid hemorrhage, history of hypertension, history of myocardial infarction, history of angina, history of migraines, history of diabetes mellitus, antiepileptic use, ruptured aneurysm location, cerebral edema, pulmonary edema, vasospasm day, baseline temperature, fever on day 8, and cerebral infarction. Dependent variable is the patient's clinical outcome (Glasgow outcome score) at 3 months.

Table 1 lists the statistically significant predictor (*P* < 0.05) variables for neurologic outcome which include normal motor response, cerebral infarction, history of myocardial infarction, cerebral edema, history of diabetes mellitus, fever on day 8, prior subarachnoid hemorrhage, admission angiographic vasospasm, neurological grade, intraventricular hemorrhage, ruptured aneurysm size, history of hypertension, vasospasm day, age, and mean arterial pressure.

All significant prognostic variables, with the exception of normal motor response, point to poorer prognosis. Presence of normal motor response at presentation signifies more favourable outcome. Its collinearity diagnostics reveal close correlation with other prognostic variables, namely, neurological grade.

Vasospasm day is closely correlated with other prognostic variables, namely, clinical and angiographic vasospasm.

Closer examination of the tirilazad patient dataset, using nontransformed data points, reveals that heteroscedasticity is present in the model. As heteroscedasticity is present, we cannot be confident that the strength of prediction of the linear regression equation from this multiple linear regression model is equally strong across all levels of the included independent variables.

Therefore, artificial neural networks were used to explore presence of complex nonlinear relationships and latent variables inherent in the database.

### 4.2. Bayesian Analysis

Beta coefficients of prognostic variables generated with Bayesian regression (WinBUGS version 1.4.3) using uninformed priors are similar in magnitude to those generated from frequentist multiple regression analysis, as demonstrated in Table 2.

Bayesian meta-analysis synthesizes research evidence from multiple independent studies. It has a hierarchical structure, placing one layer of sampling above another, with each study population having an observed odds ratio estimated from its sample of subjects. Bayesian meta-analysis models created using WinBUGS version 1.4.3 for this study gave generalizable posterior distributions of consensus odds ratios with representative medians, standard deviations, and 95% credible intervals, for the prognostic variables age [15–21], neurological grade [16–20, 22, 23], and aneurysmal size [17, 19, 23–27]. Results of hierarchical meta-analysis are shown in Table 3.

These values are very useful clinically by themselves, as they can be applied to patient prognostication. They also represent the informed priors for Bayesian regression models. Because of this database's very large sample size (*n* = 3551), beta coefficients generated with Bayesian regression (uninformed priors) are similar in magnitude to those generated from Bayesian regression (informed priors).

### 4.3. Artificial Neural Networks

Artificial neural networks (ANN), created using IBM SPSS, for prognosis in aneurysmal subarachnoid hemorrhage using the Tirilazad database revealed the following features:

 Model type: Multilayer Perceptron (MLP)
 Number of layers: 3
 Layer 1: 30 input variables
 Activation function: hyperbolic tangent
 Layer 2: hidden layer, 11 hidden or latent variables
 Layer 3: 5 output nodes
 Training: 60% of sample size
 Testing: 40% of sample size
 Training algorithm: gradient descent
 Model sensitivity: area under ROC curve = 0.85

This ANN model recognizes the presence of complex nonlinear relationships between variables, not identified by multiple linear regression.

Variables, in order of magnitude of normalized importance in Table 4, include age, second stroke, myocardial infarction, temperature, mean arterial pressure, neurological grade, ruptured aneurysm size, diabetes mellitus, angina, subarachnoid clot thickness, lung edema, admission angiographic vasospasm, previous subarachnoid hemorrhage, vasospasm day, cerebral edema, vasospasm during treatment, aneurysm location, time to treatment, normal motor response, intracerebral hematoma, normal speech, day 8 temperature, gender, eye opening, migraine history, intraventricular hemorrhage, hypertensive history, anticoagulant use, seizures, and hydrocephalus.

**Figure 2:**
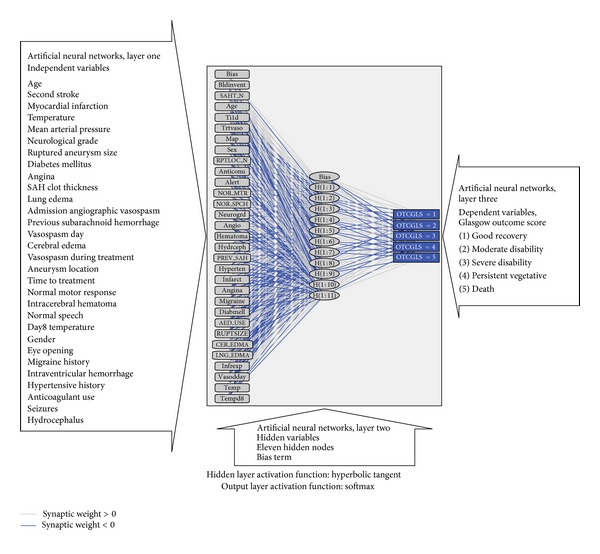
Artificial neural network output diagram with insets for each layer. Output figure generated by IBM SPSS version 19.0 (Armonk, NY, USA).

Interrelationships between input nodes (predictor variables), hidden variables (11 of them in one hidden layer), and output nodes (Glasgow outcome score) are illustrated in Figure 2. Possible latent (unobservable) variables, not measured by investigators, include the following:

(1) disrupted cerebral autoregulation contributing to both ischemia and cerebral edema after subarachnoid hemorrhage,
(2) biochemical markers of brain injury predisposing to cortical depression,
(3) cellular markers demonstrating physiologic dysfunction (such as mitochondrial dysfunction as reflected by imbalance between oxygen supply and consumption),
(4) genetic factors affecting outcome (such as inheritance of genes making patients more prone to microthrombotic events in the cerebral microvasculature disrupting cerebral blood flow),
(5) multiple drug-drug interactions, including drug hypersensitivities, especially in the elderly aneurysmal subarachnoid hemorrhage patient with multiple preexisting comorbidities,
(6) variables known to affect clinical outcome that were not captured in the Tirilazad database, including smoking and alcohol consumption,
(7) multiorgan system dysfunctions and their influence on neurologic outcome (including interactions between the central nervous system and cardiovascular, respiratory, renal, immune/hematologic, gastrointestinal (including hepatic and splenic), endocrinologic, and metabolic homeostasis).

### 4.4. Neural Networks with Bayesian Regularization

Using MATLAB version R2012a (Neural Network Toolbox), the following network was created:

 Number of layers: 3
 Layer 1: 30 input nodes (hyperbolic tangent activation)
 Layer 2: 11 hidden nodes (linear activation)
 Layer 3: 5 output nodes
 Training: 60% of sample size (2131 of 3551)
 Testing: 40% of sample size (1420 of 3551)
 Training algorithm: neural networks with bayesian regularization (“training” algorithm)

MATLAB generated the following covariance matrix from layer 2 to layer 3, representing weights, associated with their bayesian ranges, between hidden nodes and output nodes:

  [6.353 ± 1*e* − 4; 2.8199 ± 1*e* − 5; 4.5014 ± 1*e* − 5; 0.43383 ± 1*e* − 6; −3.0288 ± 1*e* − 5; −3.673 ± 1*e* − 4; −1.4082 ± 1*e* − 5; 2.2424 ± 1*e* − 5; 5.2536 ± 1*e* − 5; 2.7748 ± 1*e* − 5; 3.4145 ± 1*e* − 5].

Due to Tirilazad database's large sample size (*n* = 3551), the posterior distributions of weights from neural networks with Bayesian regularization are very narrow. Hence, choosing the mean of each distribution is the most appropriate for the calculation of each variable's normalized importance.

### 4.5. Fuzzy Logic Decision Rules

Risk factors (according to their adjusted synaptic weights from artificial neural networks and neural networks with Bayesian regularization models) can be classified into three clusters, (1) demographic (3 factors—age, time to treatment, and gender), (2) systemic (9 factors—myocardial infarction, temperature, mean arterial pressure, diabetes mellitus, angina, pulmonary edema, fever on day 8, hypertensive history, anticoagulant use), and (3) neurologic (18 factors—second stroke, neurological grade, ruptured aneurysm size, subarachnoid clot thickness, admission angiographic vasospasm, previous subarachnoid hemorrhage, vasospasm day, cerebral edema, vasospasm during treatment, aneurysm location, normal motor response, intracerebral hematoma, normal speech, eye opening, migraine history, intraventricular hemorrhage, seizures, and hydrocephalus).

### 4.6. Fuzzy Logic Rules

Individualized fuzzy logic rules can be derived based on one's experience. In this case, our three linguistic variables are (1) demographic risk factor cluster, (2) systemic risk factor cluster, and (3) neurologic risk factor cluster.

Fuzzification begins with assigning each linguistic variable a range of membership functions. When all members of each cluster are present, then maximum membership function of 1 is reached for that particular linguistic variable.

**Figure 3:**
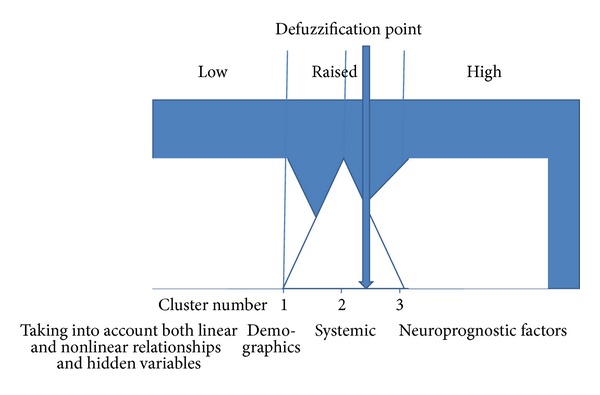
Fuzzy logic rules are applied after bayesian neural network analysis of the tirilazad database. (figure by B. W. Y. Lo).

Fuzzy inferences then proceed with derivation of “if-then” rules that define system behaviour. In our case (Figure 3), if cluster one (demographic risk factor cluster) is fulfilled, then, one has low suspicion for poor neurologic outcome. If members of both cluster one (demographic risk factor cluster) and cluster two (systemic risk factor cluster) are present, then one has raised suspicion for poor neurologic outcome. One has high suspicion for poor neurologic outcome if some members of cluster one (demographic risk factor cluster), cluster two (systemic risk factor cluster), and cluster three (neurologic risk factor cluster) are present.

Next, defuzzification step translates the linguistic variable results into the crisp control action of denoting high likelihood for poor outcome. The centroid rule is applied to designation for poor prognostication, whereby a patient fulfills risk factors from 2.5 clusters.

As an example, an elderly patient (demographic cluster), with a number of medical comorbidities (examples from systemic cluster, such as coronary artery disease, hypertensive, and diabetic), who experiences a number of neurological complications after treatment (examples from neurologic cluster, such as second stroke, cerebral ischemia, and seizures) is predicted to have a poor long-term neurologic outcome (poor three-month Glasgow outcome score).

## 5. Limitations

The techniques of bayesian neural networks with fuzzy logic inferences were applied to the Tirilazad database. We note that case mix in this database were patients who underwent surgical clipping of cerebral aneurysms. Since the conduct of the Tirilazad trials, there are advancements in both medical management and surgical treatment of cerebral aneurysms. These include improved neurocritical care of aneurysmal subarachnoid hemorrhage patients and aneurysmal coiling. In addition, the Tirilazad database did not include important prognostic variables such as smoking, alcohol consumption, rebleeding, and infection. In order to overcome these limitations, ongoing efforts are now underway to combine a number of aneurysmal subarachnoid hemorrhage databases worldwide in the multinational Subarachnoid Hemorrhage International Trialists (SAHIT) collaboration. Important prognostic variables as well as aneurysmal coiling patients will be included in this database. In addition, Bayesian neural networks with fuzzy logic inferences will be applied to the SAHIT database.

## 6. Conclusions

Complex relationships exist among heterogeneous groups of prognostic factors. The accuracy of clinical outcome prediction depends on clarification of these relationships. General linear models have been used frequently for decades. These popular techniques produce interpretable coefficients for explanatory variables and are easily estimated using commercially available statistical programs. In real life, however, data points rarely fit perfectly linear relationships. Greater deviations from linearity point to the need for exploratory analyses with complex nonlinear systems.

Typical artificial neural networks can fit training data with high precision and detect nonlinear relationships among predictor variables, with the overall aim of predicting yet to be seen observations. Neural networks also incorporate latent (unobserved variables) in one or two hidden layers. Interrelationships between independent, latent, and outcome variables are assigned synaptic weights, or connection strengths. A weighted average of these connection strengths gives a variable's normalized importance, or the percentage contribution of each predictor variable to the overall clinical outcome, taking into account the error between predicted and actual values. The sum of all relative importance values of input variables (representing influences of predictor variables on clinical outcome in relation to the rest of the independent variables) equals 100 percent. Small sample sizes can affect model building, making it more difficult to distinguish between true signal and noise. Over- and underfitting can occur in these cases, which, in turn, can affect model generalization. Neural networks with Bayesian regularization technique have been devised to overcome the above problem, whereby weights are assigned probability density distributions, incorporating Bayesian statistics to estimate weight uncertainty, or the relative degree of belief in the different values for synaptic weights.

Typical artificial neural networks give fixed structures, whereas Bayesian neural networks give flexible structures. Bayesian neural networks are ones that assign probability distributions to all elements of the network, including inputs, hidden nodes, outputs, as well as their associated weights. Prior likelihoods for probability distributions can be generated using Bayesian meta-analysis. Bayesian regularization terms are included, which prevent model over- and underfitting. In addition, Bayesian Neural Networks give generalizable posterior distributions without compromising nonlinearity properties. Bayesian Neural Networks are trained by sampling from joint posterior likelihoods of network structure and weights by Monte Carlo sampling methods. This training avoids the problem of convergence at local minima. Posterior distributions of weights can be used to evaluate uncertainties of predictions of trained networks and can also be used to assess network sensitivities. The larger the sample size, the smaller the Bayesian posterior probability distribution ranges. If data points fall into a linear relationship, typical artificial neural networks and Bayesian neural networks can detect this relationship. Hence, linear regression can be viewed as a special case of neural networks.

Typical artificial neural network and Bayesian neural network error can be due to network weight uncertainty (model uncertainty due to imperfections in data, nonoptimum network structure, and nonoptimum learning algorithms), and error from remaining sources, including intrinsic noise that includes random error due to measurement noise, and error due to finite resolution of observation system. If posterior distributions of weights are very narrow in relation to noise distribution, then the width of distribution of networks outputs can be influenced by noise. On the other hand, if the posterior distributions of weights are larger than noise distribution, then, the width of network outputs is dominated by distribution of network weights.

Fuzzy logic approach to Bayesian neural networks allows the clinician to explore where within the Bayesian range the nonlinear relationship for a particular case is most likely to exist. Results generated from Bayesian neural networks with fuzzy logic inferences will, then, slightly differ from case to case, accounting for the special characteristics of that certain case.

Fuzzy logic inferences should be applied at the end of Bayesian neural network formulation. In other words, one should allow the Bayesian neural network learning machine to do its own learning before applying fuzzy logic rules, so that all probability distributions are explored.

Bayesian neural networks with fuzzy logic inference can be conceptually interpreted as follows. Based on one's own experience (summation of existing parameters weighted by strength of belief in what happened beforehand), one can predict (based on one's assigned strength of belief) where along a spectrum of probabilities of the unknown quantity a value will end up. If it falls outside the spectrum in real life, then, one has to check whether there are still unknown elements influencing the outcome variable in question.

## Figures and Tables

**Table 1:** Multiple linear regression demonstrates significant predictor variables for neurologic outcome in aneurysmal subarachnoid hemorrhage.

Independent variable	*P* value	*β* coefficients	Collinearity diagnostics
		(95% confidence interval)	(tolerance, VIF)
Normal motor response	<0.001	−0.329 (−0.496, −0.161)	(0.27, 3.71)
Cerebral infarction	<0.001	0.790 (0.695, 0.885)	(0.86, 1.16)
History of myocardial infarction	0.009	0.386 (0.097, 0.675)	(0.92, 1.09)
Cerebral edema	<0.001	0.322 (0.190, 0.453)	(0.96, 1.05)
History of diabetes mellitus	0.028	0.239 (0.026, 0.452)	(0.98, 1.03)
Day-8 fever	<0.001	0.231 (0.150, 0.311)	(0.93, 1.08)
Prior subarachnoid hemorrhage	0.004	0.197 (0.063, 0.332)	(0.98, 1.02)
Admission angiographic vasospasm	0.015	0.175 (0.035, 0.315)	(0.93, 1.08)
Neurological grade	<0.001	0.167 (0.093, 0.242)	(0.16, 6.43)
Intraventricular hemorrhage	0.001	0.142 (0.056, 0.229)	(0.80, 1.25)
Ruptured aneurysm size	0.001	0.130 (0.053, 0.206)	(0.97, 1.03)
History of hypertension	0.009	0.119 (0.030, 0.208)	(0.85, 1.18)
Vasospasm day	0.05	0.112 (0.001, 0.225)	(0.20, 5.11)
Age	<0.001	0.018 (0.015, 0.021)	(0.86, 1.17)
Mean arterial pressure	0.012	0.003 (0.001, 0.006)	(0.91, 1.10)

VIF: variance inflation factor.

**Table 2:** Results of Bayesian regression analysis of predictors of neurologic outcome in aneurysmal subarachnoid hemorrhage using uninformed priors. Output generated by WinBUGS version 1.4.3.

Node	Mean	SD	MC error	2.5%	Median	97.5%	Start	Sample
b.MOTOR	− 0.3268	0.0833	2.606*E* − 4	−0.4904	−0.3266	−0.1632	5000	95001
b.CVA	0.9499	0.04679	1.589*E* − 4	0.8582	0.95	1.041	5000	95001
b.BSWELL	0.4307	0.06552	2.262*E* − 4	0.3021	0.4309	0.5596	5000	95001
b.MI	0.2964	0.1454	4.414*E* − 4	0.01064	0.2966	0.5805	5000	95001
b.NEUROGR	0.2762	0.02807	8.489*E* − 5	0.2213	0.2763	0.3313	5000	95001
b.DM	0.254	0.1043	3.384*E* − 4	0.04876	0.2545	0.4568	5000	95001
b.ADMITVSP	0.2417	0.06948	2.481*E* − 4	0.1046	0.2418	0.3775	5000	95001
b.PREVSAH	0.1747	0.06821	2.285*E* − 4	0.04019	0.1748	0.3073	5000	95001
b.ANSIZE	0.2124	0.03838	1.166*E* − 4	0.1373	0.2123	0.288	5000	95001
b.IVH	0.1727	0.04227	1.329*E* − 4	0.08972	0.1728	0.2551	5000	95001
b.HTN	0.1223	0.04511	1.45*E* − 4	0.03416	0.1223	0.2108	5000	95001
b.VSPDAY	0.05136	0.02759	8.475*E* − 5	−0.002518	0.05136	0.1055	5000	95001
b.AGE	0.01581	0.001591	5.454*E* − 6	0.0127	0.01581	0.01895	5000	95001
b.MAP	0.004209	0.001252	4.197*E* − 6	0.001761	0.004204	0.006663	5000	95001
b.D8TEMP	−0.08723	0.04131	1.333*E* − 4	−0.168	−0.08717	−0.006547	5000	95001

**Table 3:** Results of Bayesian hierarchical meta-analysis using WinBUGS version 1.4.3 generate posterior distributions of consensus odds ratios with representative medians, standard deviations, and 95% credible intervals, for predictor variables age, neurological grade, and aneurysm size.

Prognostic cariable	OR mean	SD	2.5%	Median	97.5%
Age	1.33	0.18	1.01	1.32	1.73
Neurological grade	2.17	0.40	1.67	2.09	3.13
Aneurysm size	1.29	0.21	1.03	1.24	1.81

**Table 4:** Results of Artificial Neural Networks reveal normalized importance values of predictor variables in aneurysmal subarachnoid hemorrhage.

Artificial neural networks independent variable	Type of prognostic factor	Importance
Age	Demographic	0.111
Second stroke	Neurologic	0.081
Myocardial infarction	Systemic	0.075
Temperature	Systemic	0.061
Mean arterial pressure	Systemic	0.054
Neurological grade	Neurologic	0.048
Ruptured aneurysm size	Neurologic	0.039
Diabetes mellitus	Systemic	0.037
Angina	Systemic	0.034
SAH clot thickness	Neurologic	0.033
Lung edema	Systemic	0.032
Admission angiographic vasospasm	Neurologic	0.029
Previous subarachnoid hemorrhage	Neurologic	0.028
Vasospasm day	Neurologic	0.028
Cerebral edema	Neurologic	0.028
Vasospasm during treatment	Neurologic	0.027
Aneurysm location	Neurologic	0.025
Time to treatment	Demographic	0.025
Normal motor response	Neurologic	0.024
Intracerebral hematoma	Neurologic	0.022
Normal speech	Neurologic	0.021
Day-8 temperature	Systemic	0.021
Gender	Demographic	0.020
Eye opening	Neurologic	0.018
Migraine history	Neurologic	0.015
Intraventricular hemorrhage	Neurologic	0.015
Hypertensive history	Systemic	0.014
Anticoagulant use	Systemic	0.014
Seizures	Neurologic	0.013
Hydrocephalus	Neurologic	0.012
